# An Investigation Into Impact of Date Seed Bioactive Compound Addition on the Quality Attributes, Shelf Life, In Vitro Digestibility, and Bioactive Properties of Cottage Cheese

**DOI:** 10.1002/fsn3.70535

**Published:** 2025-07-04

**Authors:** Jennifer Osamede Airouyuwa, Priti Mudgil, Sajid Maqsood

**Affiliations:** ^1^ Department of Food Science, College of Agriculture and Veterinary Medicine United Arab Emirates University Al‐Ain United Arab Emirates; ^2^ Water and Energy Center United Arab Emirates University Al‐Ain United Arab Emirates

**Keywords:** antimicrobial agent, antioxidant activity, bioaccessibility, functional dairy product, natural additive, total phenolic compounds

## Abstract

This study demonstrates the potential of date seed bioactive compounds (DSBCs) as a dual‐function additive in cottage cheese (CC), enhancing both its nutritional quality and shelf life. DSBCs were incorporated into CC at varying concentrations (0%, 2.5%, 5.0%, 10%, and 20%) and stored at 4°C for 14 days. Results showed a significant increase in total phenolic content (TPC) and antioxidant activity with higher DSBC concentrations, though both slightly declined during storage. Microbial analysis revealed a marked reduction in spoilage‐causing microorganisms, confirming the antimicrobial potential of DSBCs as a natural preservative. Physicochemical evaluations indicated that enriched CC retained desirable moisture, texture, and rheological properties revealed a semi‐solid cheese with a viscoelastic property. Notably, the in vitro digestion shows good bioaccessibility of DSBCs and antioxidants activity at the intestinal phase. This work highlights an eco‐innovative approach to valorizing agro‐waste, showing that the extracted DSBCs supports both as functional fortifying agent and natural antimicrobial agent that improved shelf life and consumer safety in dairy systems. The integration of DSBCs in CC represents a novel dairy innovation, offering a sustainable solution for enhancing food quality and safety.

## Graphical abstract

This study evaluated the incorporation of date seed bioactive compounds (DSBCs) into cottage cheese (CC) as a functional ingredient and natural preservative. Enrichment with DSBCs enhanced the cheese's antioxidant and antimicrobial properties, extended shelf life during 14‐day storage, and improved bioaccessibility of bioactives post‐digestion. Findings highlight DSBCs as promising functional additives for improving the nutritional quality and stability of dairy products.

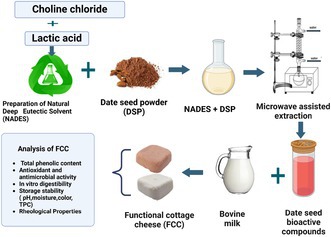

## 1 Introduction

In recent times, consumers perspective toward food have exceeded beyond satiety to improvement in the physical, mental well‐being and prevention of diseases (Liu et al. 2025; Wang et al. 2024). Studies have shown a strong correlation between diet and health; especially those associated with chronic diseases, which have led to the development of the increasing variety of fortified products such as functional food, medi‐food, vita‐food and nutraceuticals (Liu et al. 2025; Wang et al. 2024). These growing spectrum of food products contain medicinal properties with plethora bioactive compounds such as polyphenols, carotenoids and flavonoids (Arslan et al. 2020). The bioactive constituents exhibit physiological functions with potential health benefits, encompassing anti‐inflammatory, antiviral, antitumor, antioxidant, and antibacterial properties (Azarashkan et al. 2022; Cianciosi et al. 2020; Kauffmann and Castro 2023).

Date palm ( *Phoenix dactylifera* L.) is one of the most popular crops in North Africa and Middle East; it contains freshly pericarp mainly consumed as food which is a good source of simple sugars, minerals, dietary fibers, vitamins, and bioactive compounds (Jokić et al. 2019). Date fruit is processed into products like date syrup and date paste thus generating tons of date seeds as a by‐product. Date seed comprised of 10%–15% weight of date fruit (Maqsood et al. 2020), this large percentage of date seed, if not properly handled could result into environmental pollution. Studies have shown that date seed contains bioactive compounds such as flavonoids and polyphenolic compounds with myriads of anticarcinogenic, antimutagenic, antioxidant, anticarcinogenic and anti‐inflammatory properties (Ranasinghe et al. 2022).

The utilization of date seed as a value‐added and functional ingredient in food fortification supports circular bioeconomy to reduce the losses incurred from food waste disposal. Bioeconomy transforms biological resources (including agro‐waste) into useful substances like bioactive compounds and bioenergy with the aim to reduce greenhouse gases and zero waste generation (Pal et al. 2024). Most functional foods and nutraceutical products utilize agro‐industrial by‐products (seeds, leaves and pomace) as a cheap source of high value‐added ingredients which contribute positively to circular bioeconomy.

The recovery of bioactive compounds from date seeds utilizes green extraction techniques, such as microwave‐assisted extraction (MAE) coupled with natural deep eutectic solvents (NADES). These green extraction techniques are sustainable alternatives to conventional extraction methods (Radošević et al. 2018). Our previous research indicates that NADES composed of lactic acid and choline chloride show excellent extraction efficiency for date seed bioactive compounds (Airouyuwa, Mostafa, Riaz, et al. 2023a), hence the choice for its utilization. This study aimed to utilize DSBCs as functional ingredient and natural additive in cottage cheese to enhance the bioactivity and extend the shelf life of the formulated CC; offering both health benefits and improved product longevity.

To this end, varying concentrations of DSBCs were incorporated in cottage cheese and assessment of the physicochemical, antioxidant, antimicrobial, and in vitro digestion properties of the enriched CC were investigated.

## 2 Materials and Methods

### 2.1 Materials

Homogenized and pasteurized full cream milk was procured from Al Ain farm, United Arab Emirates (UAE). Date seeds (khalas variety) were obtained from Al FOAH date company, Abu Dhabi, UAE. The NADES reagents lactic acids and choline chloride and other reagent including 1,1‐diphenyl‐1‐picrylhydrazyl, Folin–Ciocalteu reagent, sodium acetate, trolox, and phenolic standards were obtained from Sigma‐Aldrich (St. Louis, MO, USA). Plate Count Agar (PCA), Potato Dextrose Agar (PDA), and Violet Red Bile Glucose Agar (VRBGA) were obtained from HiMedia Laboratories (India).

### 2.2 Date Seed Sample Preparation

The date seeds were cleaned, dried and ground using a laboratory‐scale ultra‐centrifugal mill (Retsch GmbH, Germany). A 250 μm mesh size was used to sieve the ground seeds to attain a smooth date seed powder (DSP) of similar particle size, stored at 4°C in the umber color glass bottles prior to further analyses.

#### 2.2.1 Production of Natural Deep Eutectic Solvent

The NADES was prepared by mixing 2 M concentration of lactic acid and 1 M choline chloride by continuous stirring at 80°C until a homogeneous liquid was formed (Airouyuwa, Mostafa, Renasinghe, et al. 2023b). The freshly prepared NADES was used as a green solvent for the extraction of bioactive compounds from date seed powder.

#### 2.2.2 Microwave Assisted Extraction (MAE)

The date seed bioactive compounds (DSBC) were extracted based on the previously optimization condition of MAE (Airouyuwa, Mostafa, Riaz, et al. 2023a). Detailed methodology is presented in the Supporting Information S1.1.

### 2.3 Dialysis‐Based Removal of NADES From DSBC Extracts

The extracted NADES‐based DSBC undergo further purification process by adjusting the pH to near neutral using 1 M sodium hydroxide and then the extract was dialyzed for 12 h using a dialysis bag of 3.5 kDa. The dialyzed DSBC was concentrated using freeze dryer (Zirbus, Germany) and used in the formulation of FCC. One of the ways to ascertain the removal of the DES, is through the analysis of TPC. The presence of choline chloride and lactic acid in Folin–Ciocalteu reagent gives a creaming yellow coloration when determining the TPC of date seed extract. However, with the absent of NADES in the dialyzed extract resulted in a bright yellow coloration similar to conventional solvents extracts. The dialyzed DSBC was further used as functional ingredients in cottage cheese.

### 2.4 Preparation of the Cottage Cheese (CC)

The cottage cheese was prepared with slight modification by Kapoor et al. (2021), the homogenized full cream cow milk was heated at a temperature of 80°C for 5 min, followed by the addition of vinegar and allowing it to stand for few minutes and ice cubes were added before separation of the curd from the whey using cheese cloth. The cottage cheese was formulated on replacement bases of DSBCs with the milk sample at 0%, 2.5%, 5%, 10%, and 20% (v/v) respectively; the 0% cottage cheese was without the DSBCs served as the control sample. In order to assess the physicochemical properties of the CC for 14 days, cheese samples were portioned into separate batches for each sampling day in an airtight zip lock polyethylene pouch and stored at 4°C.

### 2.5 Total Phenolic Content, Major Phenolics of DSBC and Antioxidant Activity of the DSBCs and CC

#### 2.5.1 Total Phenolic Content

The total phenolic content (TPC) of the DSBCs and the various formulations of CC were analyzed by Olatunde and Benjakul (2018). The TPC results were expressed in mg gallic acid equivalent per gram date seed powder (mg GAE/g DSP) and mg gallic acid equivalent per 100 g date seed powder (mg GAE/100 g DSP) for DSBCs and the CC respectively. The supplementary material presents a detailed methodology S1.2.

#### 2.5.2 Antioxidant Activities

The DSBCs and the CC were subjected to the DPPH radical scavenging activity as stated by Payet et al. (2005). The findings were reported in milli molars (mM) trolox equivalents per gram of DSP (mM TE/g DSP) for DSBCs and milli molars (mM) trolox equivalents per 100 g of DSP (mM TE/g DSP) for CC. The ferric reducing antioxidant power (FRAP) assay was conducted by Mostafa et al. (2022). The results were reported in similar unit as DPPH radical scavenging activity. Comprehensive methodology is provided in the Supporting Information S1.3.

#### 2.5.3 Analysis of Major Phenolic Compounds in DSBCs and CC Samples by UHPLC

An aliquot of DSBCs and CC samples were further filter through the 0.45 μm micropore membrane and transferred into 2 mL HPLC vials for quantifying the major phenolic compounds using Ultimate 3000 UHPLC system equipped with a quaternary Series pump, autosampler and photodiode array detector (Thermo Fisher Scientific, Waltham, MA, USA). The supplementary material presents a detailed technique S1.4.

### 2.6 In Vitro Simulated Gastro‐Intestinal Digestion (SGID) of DSBCs and CC

Prior to the digestion of FCC, 200 mL of the various digestive buffers comprising simulated salivary fluid (SSF), simulated gastric fluid (SGF) and simulated intestinal fluid (SIF) solutions at pH 7, 3, and 7, respectively, were prepared following the method of (Brodkorb et al. 2019). Comprehensive methodology is provided in the Supporting Information S1.5.

### 2.7 Physicochemical Properties of CC

The moisture content was analyzed by AOAC (2002). Briefly, 5 g of CC were placed in an oven at 105°C until a constant weight was achieved (Feldsine et al. 2002).

The protein content of CC was estimated by AOAC (2002) using the Kjeldahl method (*N* × 6.38). All analyses were carried out in triplicates.

The pH of the CC was determined by homogenizing 5 g of each cheese sample in 50 mL distilled water. Immediately after CC homogenization, the pH was measured using a pH meter (EUTECH pH Meter, Italy).

The color of the CC was investigated on the freshly prepared cheese, and those stored for Day 7 and Day 14 using colorimeter (Hunter lab colorFlex EZ spectrophotometer, USA) based on color coordinates L* represent dark to light values, a*‐represent red to green values and b*‐represent yellow to blue values.

#### 2.7.1 Textural Properties of CC

The texture analysis of CC was carried out following the methods reported by (Jia et al. 2022) with slight modifications. Comprehensive methodology is provided in the Supporting Information S1.6.

#### 2.7.2 Rheological Properties of CC

The rheological properties of the CC enriched with DSBC were evaluated using a Discovery Hybrid Rheometer (TA Instruments, Delaware, USA) according to the method of Azarashkan et al. (2022), with slight modification. Comprehensive methodology is provided in the Supporting Information S1.7.

#### 2.7.3 Fourier Transform Infrared (FTIR) Spectroscopy of CC

The FTIR spectrum of the CC was evaluated on the freeze‐dried cheese sample in order to eradicate the interference of water from all the spectra. The freeze‐dried CC was subjected to Spectrum Two FT‐IR Spectrophotometer (PerkinElmer, UK) with a wavelength of 4000 to 400 cm^−1^, and at a resolution of 4 cm^−1^.

### 2.8 Microbiological Analyses

Ten grams of each sample comprising the cheese enriched with DSBCs were aseptically dispersed in a stomacher bag and dissolved in 90 mL of sterilized 0.1% peptone water. The microbiological assays were carried out by the pour plating methods to determine total bacterial count using Plate Count Agar (PCA) and plates were incubated for 48 h at 37°C. Yeast and mold were determined using Potato Dextrose Agar/Chloramphenicol, and plates were incubated for 5 days at 27°C. Although Violet Red Bile Glucose Agar (VRBGA) was used to determine the growth of coliforms, for which the plates were incubated for 48 h at 37°C. The bacterial colonies were counted using Scan 1200 (Interscience, UK) and the results were expressed as log CFU/g of CC.

### 2.9 Sodium Dodecyl Sulphate‐Polyacrylamide Gel Electrophoresis (SDS‐PAGE) of CC

SDS‐PAGE of FCC was carried out by following a previous method described (Mudgil et al. 2019) with slight modifications. Comprehensive methodology was provided in the Supporting Information S1.8.

### 2.10 Statistical Analysis

Five treatments of CC were prepared in two replicates and all the analyses conducted on the cheese were performed in triplicates. The data were then subjected to one‐way ANOVA and Tukey's multiple range test was used to perform suitable means differences using SPSS software (Version 23) in order to identify statistically significant differences among samples and between each treatment (*p* < 0.05).

## 3 Results and Discussion

### 3.1 Phenolic Content and Antioxidant Activity of DSBCs and CC

**FIGURE 1:**
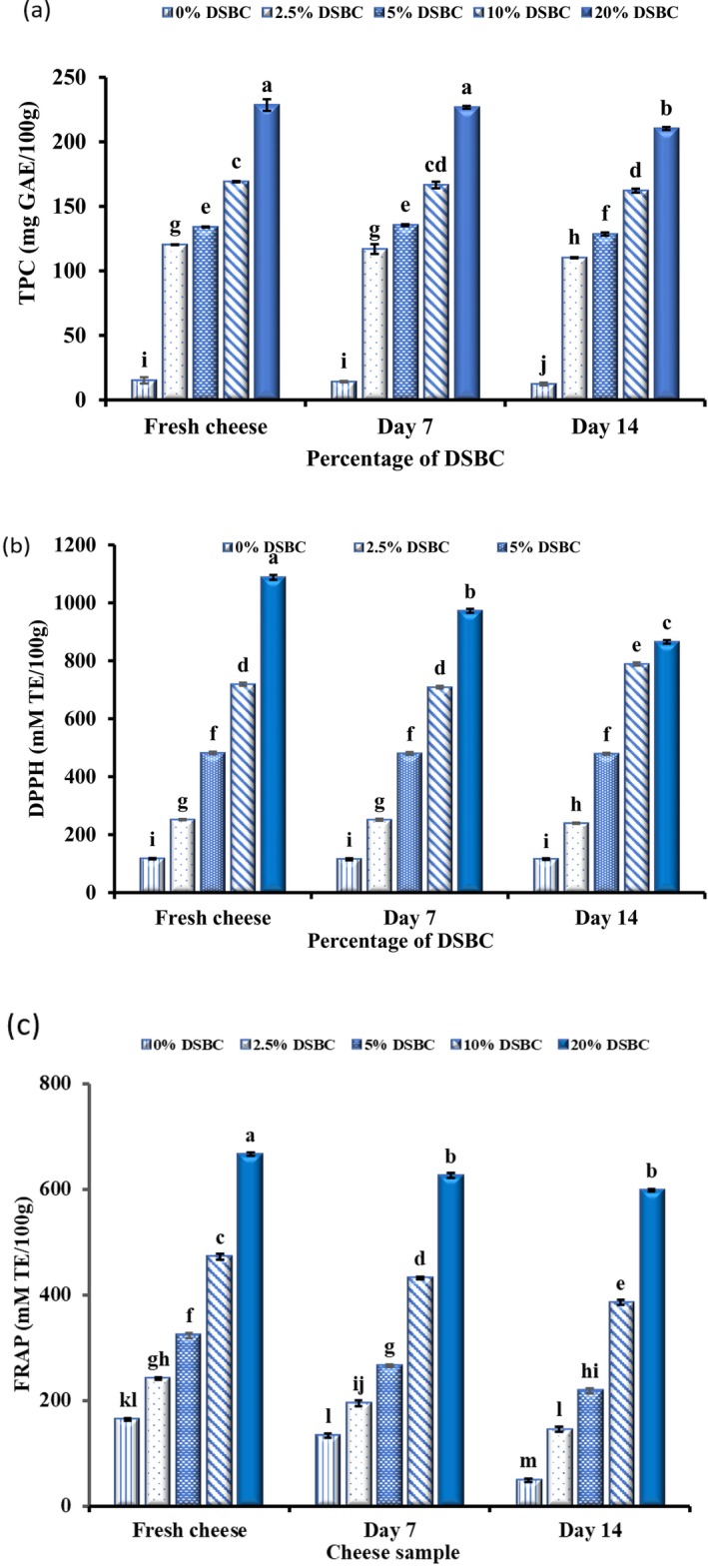
Total phenolic content (a) and antioxidant activity (DPPH radical scavenging activity (b) and FRAP (c)) of cottage cheese during 14 days of refrigerated storage.

The TPC and antioxidant activity (DPPH and FRAP) of the extracted DSBC were 153.58 ± 72 mg GAE/g DSP, 601.89 ± 83 mM TE/g DSP and 787.53 ± 45 mM TE/g DSP, respectively, as shown in Supporting Information S2.1. The cottage cheese enriched with DSBCs reflected a significant increase (*p* < 0.05) in the cheese phenolic content and antioxidant activity; an indication that date seed is an excellent source of bioactive compounds. Five different formulations were prepared with 2.5%–20% DSBC and control samples (0%) without DSBC. Increase in the DSBC in the enriched CC from 2.5% to 20% (80.3 ± 0.25–228.53 ± 4.53 mg GAE/100) resulted to corresponding increase in the TPC of the cheese as shown in Figure 1a. Although there is minimal quantity of phenolic content present in the control samples (15.11 ± 0.44 mg GAE/100 g) which could be attributed to the presences of some antioxidant peptides detectable by Folin‐Ciocateu reagent (Darwish et al. 2020). The antioxidant activity of the cheese was estimated by DPPH and FRAP assays as shown in Figure 1b,c. The fortified cheese reflected significantly higher (*p* < 0.05) DPPH activity than the control samples (0% DSCB), with 20% DSBC exhibited the highest DPPH activity (1088.41 ± 8.5 mM TE/100 g) and FRAP (666.14 ± 3.15 mM TE/100 g) values than other formulations. The TPC of the fortified cottage cheese during the storage at 4°C were relatively stable after 7 days storage, however, there were significantly decreased in the TPC at 14 days storage (*p* < 0.05). The stability of polyphenolic compounds in the CC could be as a result of the binding of phenolic compounds to casein proteins via noncovalent interactions, including hydrogen bonding, hydrophobic effects, and π–π stacking, as similarly observed in protein–polyphenol systems such as those involving β‐lactoglobulin (Sun et al. 2022; Li et al. 2023). These interactions may influence the protein matrix structure, enhancing phenolic stability. Moreover, casein–polyphenol complexes can reduce oxidative reactions within the matrix, improving both shelf life and nutritional quality. Hurtado‐Romero et al. (2023), reported the fortification of symbiotic petit cheese with blueberry as functional ingredient, the study revealed that the bioactive compounds were stable up to 14 days, after which it slightly decreased during the 21 days storage period. The fortification of DSBCs in CC serves as a good matrix for delivery polyphenols as functional ingredient owning to the interactions between polyphenol and casein network.

### 3.2 In Vitro Digestibility and Bioaccessibility of CC

**FIGURE 2:**
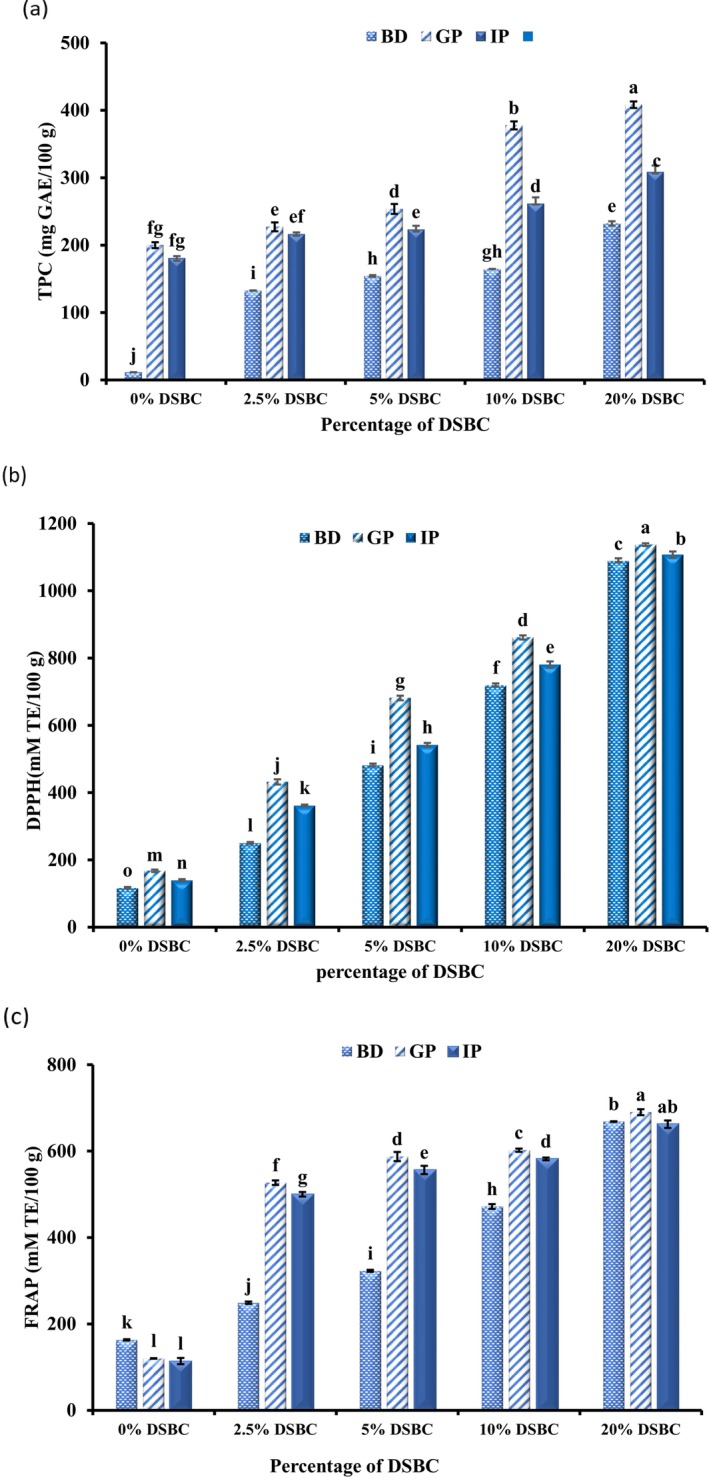
Effect of in vitro digestibility of the fortified cottage cheese on (a) Total phenolic content, (b) DPPH radical scavenging activity, and (c) FRAP. BD: before invitro‐digestion; GP: gastric phase digestion; IP: intestinal phase digestion.

In vitro gastro‐intestinal digestion of the enriched CC with DSBC was investigated, and the results are shown in Figure 2. The TPC of all cheese samples at the gastric phase showed a profound increase compared with the TPC before digestion (*p* < 0.05) as shown in Figure 2a. The results revealed that the low pH of the gastric digestion and action of digestive enzymes enhanced the release of phenolic compounds. In addition, the acidic pH of the gastric phase facilitated the disruption of the casein network and other macromolecules, thus enhancing the release of the bound polyphenols. However, at the intestinal phase, there was a slight decrease in the phenolic content of the CC compared to the gastric phase. This slight degradation of TPC in the intestinal phase, when compared to the gastric phase digestions, could be a result of the transition from acidic pH in the gastric phase to alkaline pH in the intestinal phase. This reported a 71% increase in total polyphenol of green tea cheese at the gastric phase, while an 18% decrease was observed at the end of the intestinal phase digestions. Similarly, Tagliazucchi et al. (2010) reported an approximately 15% loss of polyphenol during the transition from the acidic gastric phase to the mild alkaline intestinal phase in the digestion of grape polyphenols. In general, TPC at the intestinal phase was higher than the TPC of the cheese sample before digestion. The results showed that the entrapment of phenolic compounds in the cheese matrix and their interaction with the amino acid residues of the casein moiety may delay the release of phenolic compounds in the gastric phase. Eventually, the total phenolic compounds released after digestion of the CC were significantly higher than before digestion, which is an indication of enhanced bioaccessibility of the phenolics at the gastrointestinal phase (Aalim et al. 2024).

The in vitro digestion model provides an insight to the bioaccessibility of antioxidants and phenolics in biological systems. In this study, the changes in antioxidant activities (DPPH and FRAP) of CC upon simulated in vitro gastrointestinal digestion are presented in Figure 2b,c. Overall, the antioxidant activity was quite stable during the digestion process, with a slight increase in the intestinal phase when compared to the undigested cheese samples. The antioxidant activity of CC at 20% DSBC before digestion was 1088.0 ± 8.1 and 668.37 ± 5.7 (Mm TE/100 g DSP) for DPPH and FRAP, respectively, whereas after the gastrointestinal phase, there was a significant increase in the antioxidant activity, reaching 1107 ± 9.5 and 677.35 ± 8.6 (Mm TE/100 g DSP) for DPPH and FRAP, respectively (*p* < 0.05). The increase in the antioxidant activity of CC upon in vitro digestion, could be due to the release of both bioactive compounds and bioactive peptides from the DSBCs and casein moiety by the action of digestive enzymes (Helal and Tagliazucchi 2018). The co‐digestion of DSBC with milk proteins may produce synergistic effects, leading to enhanced antioxidant outcomes, as supported by the mechanistic principles discussed by Liu et al. (2025) who study influence on the anti‐inflammatory activity of soy peptides during digestion and absorption in different enzymatic hydrolysis conditions. This dual contribution of protein‐derived peptides and released phenolics from DSBC highlights the functional potential of this fortified dairy system.

### 3.3 Identification of Individual Phenolic Compounds in CC

The major phenolic compounds in the DSBC extract and fortified cheese samples before and after in vitro gastrointestinal digestion were evaluated, and the results are presented in Table 1. Twelve individual phenolic compounds were present in the DSBC extract which includes gallic acid, protocatechuic, caffeic acid, rutin hydrate, catechin, syringic acid, coumaric acid, quercetin, benzoic acid, vanillic acid, 4‐hydroxybenzoic acid, and 1, 2, −dihydroxybenzoic acid; with caffeic acid, catechin and quercetin were predominantly present in the extract. Two additional compounds, chlorogenic acid and ferulic acid, were not detected in the DSBC extract, but were detected in trace amounts in the CC which could be from the milk samples. The phenolics detected in cheese samples before digestion in trace amount were gallic acid, protocatechuic acid, caffeic acid, rutin hydrate, and chlorogenic acids, while others were detected in large quantities. According to Ozdal et al. (2019), phenolic compounds have a high propensity for binding to milk sample, and the affinity of polyphenols binding with milk sample increases, especially after heat treatment, due to the formation of bound micelle complexes (Kostić et al. 2021). In addition, the tendency of the binding between phenolics and milk is highly dependent on the three‐dimensional structure of protein–polyphenol interactions (Jing et al. 2020; Li et al. 2023). On the other hand, some phenolic compounds that were not present in the undigested CC, however, were detected in the cheese after gastrointestinal digestion, which could be attributed to the fact that some phenolics were entrapped in the casein matrices upon enzyme hydrolysis and pH changes in the gastrointestinal environment led to their release (Liu et al. 2025). In addition, coumaric acid and quercetin were not detected after the gastrointestinal phase, which may explain the formation of complex phenolic derivatives through auto‐oxidation, polymerization, and epimerization (Kostić et al. 2021). Similarly, there was a significant degradation (*p* < 0.05) observed in 4‐hydroxybenzoic acid after in vitro digestion compared to that before digestion in all FCC samples. Furthermore, 1,2‐dihydroxybenzoic acid, catechin, vanillic acid, syringic acid, and ferulic acid tend to be more stable with increased after in vitro digestion as compared to the same sample before digestion. These compounds could also contribute to the slight increase in the TPC, DPPH and FRAP yields observed after digestion, as indicated in Figure 2. In general, the phenolic compounds recovered after in vitro digestion were higher than the phenolic compounds of undigested cheese, indicating that CC is a good vehicle for delivery of DSBC with good bioaccessibility of the phenolics in the gastrointestinal phase.

**TABLE 1:** The UHPLC analysis of major phenolic compounds in the cottage cheese enriched with date seed bioactive compounds (DSBCs) before and after in vitro digestion.

Before invitro‐digestion of cheese sample						After invitro‐digestion of cheese sample			
Phenolics (mg/100 g DSP)	DBSC extract	2.50% DBSC	5% DBSC	10% DBSC	20% DBSC	2.50% DBSC	5% DBSC	10% DBSC	20% DBSC
Gallic acid	0.95 ± 0.2^a^	nd	nd	nd	nd	0.4 ± 0.2^b^	0.3 ± 0.2^b^	0.07 ± 0.2^c^	nd
Protocatechuic acid	0.9 ± 0.2c	nd	nd	nd	nd	4.2 ± 0.2^a^	2.2 ± 0.2^b^	1.6 ± 0.9^bc^	nd
Caffeic acid	6.1 ± 1.0^c^	nd	nd	nd	0.7 ± 0.1^c^	39.±0.9^a^	33.4 ± 1.6^a^	30.7 ± 0.8^a^	17.7 ± 0.9^b^
Rutin hydrate	1.0 ± 0.2^b^	0.3 ± 0.1^c^	nd	nd	nd	2.7 ± 0.6^a^	2.8 ± 0.1^a^	2.9 ± 0.1^a^	0.9 ± 0.0^b^
Catechin	4.4 ± 0.1^e^	2.8 ± 0.1^e^	3.4 ± 0.2^e^	3.4 ± 0.2^e^	1.6 ± 0.0^e^	31 ± 0.2^d^	35 ± 0.2b^c^	38.9 ± 2.2^b^	49.1 ± 1.2^a^
Syringic acid	3.1 ± 0.8^e^	0.7 ± 0.0^e^		0.3 ± 0.2^e^	0.4 ± 0.1^e^	9.3 ± 0.2^d^	10.8 ± 0.2^c^	15.21^b^	16.9 ± 0.1^a^
Coumaric acid	2.2 ± 0.1^a^	0.1 ± 0.2^b^	1.8 ± 0.2^a^	0.3 ± 0.2^b^	2.2 ± 0.3^a^	nd	nd	nd	nd
Ferulic acid	nd	1.3 ± 0.2^cd^	0.4 ± 0.2^e^	0.5 ± 0.2^e^	1.5 ± 0.2^c^	2.2 ± 0.3^c^	3.4 ± 0.2^b^	5.8 ± 0.6^a^	5.1 ± 0.2^a^
Chlorogenic acid	nd	nd	0.8 ± 0.0^a^	nd	0.3 ± 0.2^a^	0.7 ± 0.1^a^	0.58 ± 0.3^a^	0.3 ± 0.1^a^	0.5 ± 0.2^a^
Quercetin	4.0 ± 1.0^a^	1.2 ± 0.0^cd^	2.4 ± 0.5^bc^	1.8 ± 0.2^c^	3.1 ± 0.8^ab^	nd	nd	nd	0.3 ± 0.2^d^
Benzoic acid	3.8 ± 0.8^c^	3.5 ± 0.1^c^	4.80 ± 0.8^c^	7.1 ± 0.2^b^	6.5 ± 0.5^b^	7.2 ± 0.8^b^	nd	nd	17.3 ± 1.2^a^
Vanillic acid	0.4 ± 0.2^b^	0.1 ± 0.0^b^	0.2 ± 0.2^b^	0.2 ± 0.2^b^	0.2 ± 0.0^b^	1.1 ± 0.2^b^	5.6 ± 0.4^a^	6.0 ± 0.5^a^	4.5 ± 0.2^a^
4‐Hydroxybenzoic acid	2.1 ± 0.3^a^	1.3 ± 0.7^abcd^	nd	1.8 ± 0.2^abc^	1.9 ± 0.2^ab^	0.2 ± 0.2^e^	0.6 ± 2^de^	0.9 ± 0.3^cde^	1.1 ± 0.2^bcde^
1,2,‐Dihydroxybenzoic acid	14.4 ± 0.9^a^	1.8 ± 0.2^d^	0.5 ± 0.2^e^	1.0 ± 0.2^de^	1.2 ± 0.2^de^	8.7 ± 0.2^c^	10.2 ± 0.6^b^	13.3 ± 1.6^a^	9.0 ± 0.4^bc^

*Note:* The lower‐case letters represent the significant difference among the samples (*p* value < 0.05).

### 3.4 Physicochemical Properties of CC

The physicochemical characteristics of the enriched cottage cheese (CC) with date seed bioactive compounds are presented in Table 2. The incorporation of DSBC in the CC significantly influenced (*p* < 0.05) the pH, moisture content, and protein content. The addition of DSBC in the cheese significantly increased the moisture content of the fortified cheese as the concentration of DSBC increased (*p* < 0.05). However, during the 14 days storage period, the moisture content of the CC slightly decreased, which could be attributed to the evaporation of moisture from the cheese surfaces (Pérez‐Soto et al. 2021) addition, the pH values of the CC decreased as the incorporation of DSBC concentration increased. The highest pH value found in the 0% DSBC (control cheese samples) was 5.56 ± 0.14, and the lowest pH value was 4.51 ± 0.11 of cheese samples fortified with 20% DSBC. The decrease in the pH values of the CC could be attributed to the effect of phenolic acids present in the DSBCs. A similar finding was observed in the study of Du et al. (2021), who reported a decrease in the pH value of yogurt fortified with mulberry powder due to the presence of phenolic acids. In addition, the pH values of all the cheese samples slightly decreased during the storage period of 14 days; however, this decrease in the pH values was not significant (*p* > 0.05). The slight decrease in the pH values could be attributed to the interaction of proteins and the hydrolyzed minerals during the storage period (Pastorino et al. 2003; Darwish et al. 2020). The pH values in all the fortified cheese samples slightly decreased, which was in line with the study of Darwish et al. (2020); Tawfek (2018). The protein content of the control cheese sample was higher compared to all the fortified cheese (*p* < 0.05). The protein content of the cheese dropped when the concentration of DSBCs in the CC increased from 2.5% (21.78 ± 0.03) to 20% (19.97 ± 0.45). This could be attributable to the fact that the CC was fortified with DSBC on a replacement basis. In general, the physicochemical properties of the formulated CC were highly influenced by the incorporation of DSBCs.

**TABLE 2:** Comparison of color, pH, and moisture content of the date seed bioactive compounds enriched cottage cheese at 4°C for 14 days.

Samples	Storage period	L*	a*	b*	Moisture content	pH	Protein content
Control	Fresh	88.15 ± 1.36^a^	−1.34 ± 0.18^g^	10.41 ± 0.49^fg^	48.93 ± 0.80^ef^	5.56 ± 0.14^a^	24.68 ± 0.53a
	Day 7	83.27 ± 1.24^b^	−1.10 ± 0.15^g^	10.13 ± 0.65^g^	47.63 ± 0.50_f_	5.41 ± 0.08^ab^	
	Day 14	80.37 ± 0.97^cd^	−1.16 ± 0.28^g^	9.56 ± 0.42^g^	47.14 ± 0.85^f^	5.35 ± 0.05^ab^	
2.5% DSBC	Fresh	82.47 ± 1.40^bc^	4.03 ± 0.69^f^	10.56 ± 0.76^fg^	53.79 ± 0.27^abc^	5.26 ± 0.22^abc^	21.78 ± 0.03b
	Day 7	78.52 ± 0.69^d^	4.13 ± 0.71^f^	10.52 ± 0.71^fg^	52.89c±0.43^abcd^	5.16 ± 0.21^abc^	
	Day 14	70.62 ± 1.22^f^	4.64 ± 0.28^f^	10.09 ± 0.16^g^	51.74 ± 0.08^bcd^	5.05 ± 0.23^bcde^	
5% DSBC	Fresh	75.40 ± 2.38^e^	7.23 ± 0.91^e^	11.95 ± 0.53^de^	53.80 ± 0.87^abc^	4.77 ± 0.08^cdef^	21.16 ± 0.01c
	Day 7	74.26 ± 2.37^ef^	7.41 ± 040^e^	11.82 ± 0.12^de^	53.82 ± 0.70^abc^	4.74 ± 0.12^def^	
	Day 14	65.05 ± 0.43^g^	7.33 ± 0.41^e^	11.36 ± 0.61^ef^	51.75 ± 0.08^cd^	4.53 ± 0.09^f^	
10% DSBC	Fresh	66.03 ± 1.12^g^	11.50 ± 0.36^c^	13.61 ± 0.04^b^	53.88 ± 0.14^abc^	4.55 ± 0.07^ef^	20.44 ± 0.10d
	Day 7	66.55 ± 0.05^g^	11.23 ± 0.42^c^	12.74 ± 0.05^cd^	53.76 ± 1.36^abc^	4.50 ± 0.01^f^	
	Day 14	57.50 ± 0.09^h^	10.14 ± 0.01^d^	12.71 ± 0.22^cd^	52.30 ± 0.47b^cd^	4.34 ± 0.06^f^	
20% DSBC	Fresh	60.45 ± 0.57^h^	14.88 ± 0.21^a^	14.82 ± 0.27^a^	54.91 ± 1.11^a^	4.51 ± 0.11^ef^	19.97 ± 0.45d
	Day 7	59.44 ± 0.15^h^	14.45 ± 0.16a^b^	14.45 ± 0.11^ab^	54.56 ± 0.49^ab^	4.49 ± 0.11^f^	
	Day 14	52.21 ± 0.49^i^	13.63 ± 016^b^	13.23 ± 0.27^c^	53.86 ± 0.12^abc^	4.33 ± 0.04^f^	

*Note:* The mean value represents (*n* = 3) ± SD. The lower‐case letters in each column represent the significant difference among the various samples (*p* value < 0.05).

#### 3.4.1 Color of CC

Considering the high phenolic content and antioxidant activity present in DSBC, its utilization as a functional ingredient in cottage cheese could serve as a cheap and affordable means of production of fortified cottage cheese (Al‐Khalili et al. 2023). The color analysis of the CC was studied in freshly prepared cheese and in samples stored for 7 and 14 days at refrigerated temperature. The results of the color evaluation are shown in Table 2. The L* values indicate lightness‐darkness, with higher L* values indicating a clearer and whiter cheese samples. The control sample showed significantly higher L* values compared to other enriched cheese samples (*p* < 0.05). As the concentration of DSBC increased from 2.5%–20%, the lightness (L*) values significantly decreased (*p* < 0.05). The decrease in L* values observed in the fortified cheese could be attributed to the increasing concentration of DSBCs from 2.5% to 20%, which interfered with the cheese color, resulting in lower L* values. This finding is similar to previous studies, where the incorporation of bioactive functional ingredients such as *Foeniculum vulgare* Mill. (fennel) decoction to cottage cheese (Caleja et al. 2015), chia seed extract to ricotta cheese (Hosseini et al. 2023) and date syrup to processed cheese (El‐Loly et al. 2021) resulted in a decrease in the L* values. The L* lightness value of the control and fortified cheese significantly decreased during the 14 days of storage (*p* < 0.05). These observations were similar to the study of El‐Loly et al. (2021) who incorporated date syrup into processed cheese. The decrease in L* values could be due to the Maillard reaction occurring between lactose and proteins present in the cheese matrix (El‐Loly et al. 2021). The a* and b* parameters are an indication of the redness to greenness and blueness to yellowness tendency, respectively. The control cheese samples had negative a* values, which is an indication of the greenness spectrum; the addition of DSBC to the cottage cheese changed the spectrum from negative to positive a* values. As the quantity of DSBC incorporated in the cheese sample increased from 2.5% to 20%, there was a significant increase (*p* < 0.05) in the a* values. The b* values of the control and the enriched CC had positive values, an indication of cheese yellowness. There was no change in the b* values of 0% and 2.5% CC; however, as the percentage of DSBC incorporated in the CC increased beyond 2.5%, the yellowness of the cheese slightly increased (*p* < 0.05). No changes were observed in the a* and b* values of the control samples and those of low concentrations of the enriched CC (2.5% and 5% of DSBC) during the storage period; however, as the concentration of DSBC increased in the CC from 10% to 20%, there were significant decreases (*p* < 0.05) in the a* and b* of the fortified cheese, which could be due to slight degradation of the DSBC during the 14 days storage period.

#### 3.4.2 Textural Properties of CC

The textural properties of any cheese are significantly influenced by its composition, such as moisture content, fat content, protein content, pH, salt, and other functional ingredients/additives (DSBC). Table 3 shows the textural properties of CC (hardness, adhesiveness, springiness and resilience). Control cheese (0% DSBCs) had significantly higher hardness, which was positively influenced by its compositions, such as significantly lower moisture content, higher protein content, and relatively higher pH compared to the fortified cheese. As the percentage of DSBCs incorporated in the CC increased, there was a significant decrease in the hardness of the fortified cheese (*p* < 0.05). In addition, protein content plays a major role in the textural properties of cheese; casein, being the major protein found in the cheese, has the ability to directly form a honeycomb network with the calcium bridge. This structural casein network provides the matrix for salt, moisture, and fat (Zheng et al. 2016) resulting in its unique textural properties. The replacement of a certain portion of protein with DSBC could interfere with and disrupt the casein structural network, which results in a reduction in the hardness of the CC; this could also be related to the high moisture content observed in the enriched CC at higher formulations. Previous studies have also documented a decrease in the textural properties of cheese upon addition of polyphenolic extract. Giroux et al. (2013) reported that the fortification of cheese with tea extract rich in bioactive compounds has the potential to reduce the fortified cheese's elasticity and cohesiveness due to alteration in the paracasein structural network. Similarly, the supplementation of bovine milk cheese with orthodox black tea reduced the cheese's hardness (Fadhlurrohman et al. 2023). The work required to overcome the attractive force between the cheese and the surface or material it is adhered to is described as adhesiveness (Zheng et al. 2016). Food taste and flavor release in the consumption of cheese is associated with cheese adhesiveness. However, too high adhesiveness may result in stickiness of cheese to the package. One of the major factors contributing to cheese adhesiveness is its fat content. An increase in fat content and reduction in protein content allows the cheese to easily melt, resulting in an increase in the cheese's adhesiveness (Zheng et al. 2016). Although the fat content of CC was not investigated in this study, the control samples and 2.5% CC had significantly higher adhesiveness. However, at higher formulations of DSBC in cheese, there was a significant reduction (*p* < 0.05) the adhesiveness of the CC. The lower adhesiveness of enriched cheese with 5%–20% DSBC could be attributed to the substitution of milk with a higher level of DSBC, resulting in a reduced protein content and providing a more lubricating or adhesive effect to the cheese since it was formulated on a replacement basis. Springiness, on the other hand, is the rate at which the cheese returns to its original form after compression between the textural probe and its surface where the cheese is placed. The springiness of 0% CC was significantly higher than that of fortified cheese samples (CC). The result herein revealed that the incorporation of DSBC into the fortified cheese had a profound influence on the cheese's textural properties, especially at higher concentrations.

**TABLE 3:** Textural properties of the cottage cheese enriched with date seed bioactive compounds (DSBCs).

Samples	Hardness (g)	Adhesiveness (g)	Springiness	Resilience
Control	4237.25 ± 2.47^a^	78.50 ± 0.71^a^	0.47 ± 0.01^a^	0.16 ± 0.01^b^
2.5% DSBC	4102.00 ± 2.12^b^	61.00 ± 1.41^b^	0.37 ± 0.01^b^	0.16 ± 0.00^b^
5% DSBC	3875.25 ± 6.72^c^	16.75 ± 0.35^c^	0.40 ± 0.00^b^	0.24 ± 0.01^a^
10% DSBC	3736.00 ± 8.49^d^	6.25 ± 0.35^d^	0.39 ± 0.01^b^	0.23 ± 0.00^a^
20% DSBC	3588.25 ± 0.35^e^	4.50 ± 0.00^e^	0.20 ± 0.00^c^	0.23 ± 0.00^a^

*Note:* The mean value represents (*n* = 3) ± SD. The lower‐case letters in each column represent the significant difference among the various samples (*p* value < 0.05).

#### 3.4.3 Cheese Rheological Properties

**FIGURE 3:**
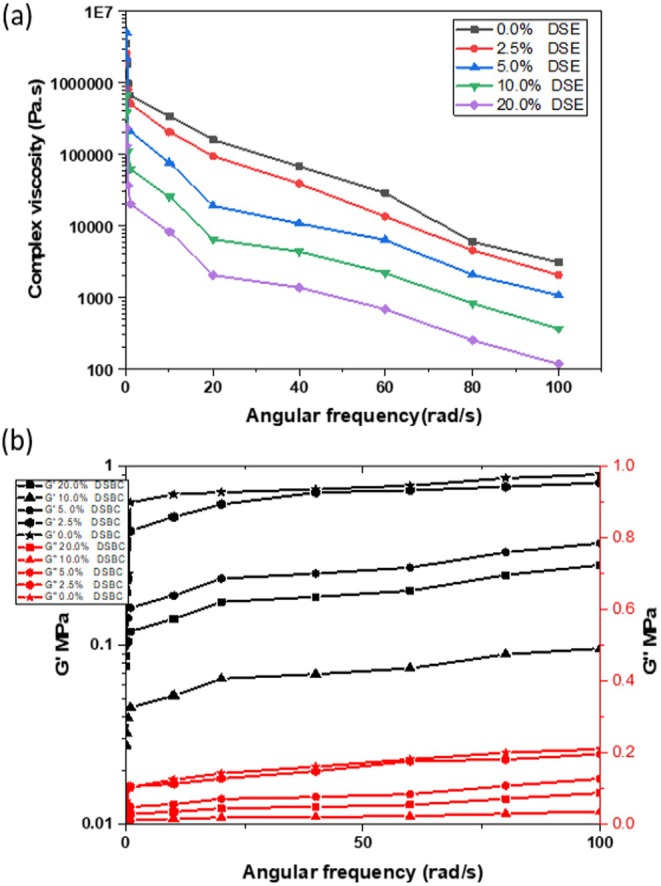
(a) The complex viscosity and (b) storage modulus (*G*′) and loss modulus (*G*″) of functional cottage cheese fortified with different concentration of DSBC using the frequency sweep test (0 to 100 rad/s).

To observe the rheological properties, cheese was subjected to stress, and the deformation under stress was observed using different rheological parameters. Cheese can be classified as an ideal viscous liquid, elastic solid, or viscoelastic solid depending on the level of deformation (Singh et al. 2023). Figure 3a represents the complex viscosity (*ƞ) under the frequency sweep of the CC samples. The complex viscosity of all the cheese samples decreased with the increase in the angular frequency, which implies that the cheese samples possess a thixotropic behavior. Azarashkan et al. (2022) and Hesarinejad et al. (2021) reported a decrease in the complex viscosity as the angular frequency increased in ricotta cheese. As the concentration of DSBCs in the fortified cheese increased, the cheese hardness decreased, and moisture content increased, as shown in Tables 1 and 2, which consequently reduced the complex viscosity, owing to the fact that the complex viscosity is a function of the total hardness of a given cheese sample. Furthermore, the values of storage modulus *G*′ and loss modulus *G*″ showed a positive correlation as the angular frequency increased from 1% to 100 rad/s; both *G*′ and *G*″ also increased. According to Černíková et al. (2017) and Gabriele et al. (2001) the increase in the values of *G*′ and *G*″ corresponds well to the increasing gel strength of foodstuffs and semi‐solid materials. The *G*′ and *G*″ of the CC increased as the angular frequency increased, with a noticeably higher increase in the *G*′, which indicated an elastic nature and solid behavior of the CC. It is pertinent to note that a higher concentration of DSBC in the CC lowered the solid behavior of the cheese, as indicated in the reduced *G*′ values, as shown in Figure 3b. The CC exhibited viscoelastic behavior with higher *G*′ compared to *G*″ values. In addition, at higher formulations (10% and 20% DSBC) the presence of higher DSBCs influenced the cheese's rheological property with softer cheese and significantly higher moisture content compared to lower formulations and control samples.

#### 3.4.4 Fourier Transform Infrared (FTIR) Spectroscopy of CC

**FIGURE 4:**
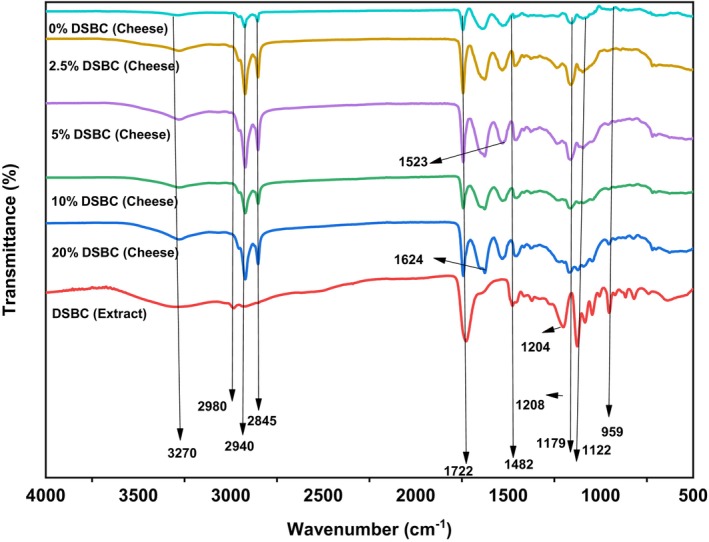
Fourier transform infrared (FTIR) spectroscopy of the various formulated cottage cheese fortified with different levels of date seed bioactive compounds (DSBCs).

The FTIR of the DSBC extract (red spectrum) and the enriched cheese (blue, green, purple, brown and turquoise representing 20%, 10%, 5%, 2.5%, and 0% CC formulations) were represented in Figure 4. The spectra ranging from 3500 to 3100 represents the –OH bands stretching obtained from the different chemical components of the phenolic extracts, and cheese (Bro and Smilde 2014; Grasel et al. 2016). Although, the FTIR of all the cheese samples both control and fortified cheese did not show a long stretch vibration at the –OH regions unlike the study of Foda et al. (2013) who investigated the FTIR of soft white cheese. The reason for the short bands observed in our findings could be attributed to the fact that the samples utilized for FTIR analysis were freeze dried cheese samples to eliminate most of its water. The long stretches observed in DSBC extract spectrum represent the polymeric hydroxyl group and the hydrogen bond of the polyphenolic compounds (Wongsa et al. 2022). In the region 3000–2800 cm^−1^ absorption bands represent the symmetric and the asymmetrical vibration of C‐H which could be derived from the cheese fatty acid (Foda et al. 2013) and sugars from the DSBC extract (Aslan 2013; Grasel et al. 2016). Low intensity was observed in the control sample spectrum unlike the fortified cheese with higher intensities and longer bands stretch observed between the region 3000–2800 cm^−1^ could be due to hydrophobic interactions between the polyphenols ((+)‐catechin) from the DSBCs and fat globules membranes of the milk sample (Rashidinejad et al. 2016). In the region corresponding to 1750–1650 cm^−1^ represents C=O of acids and esters which could be fatty acid from milk (Foda et al. 2013), and phenolic acids, especially esters from hydrolysable tannins a derivative of gallic acid (Aslan 2013; Grasel et al. 2016). The wavenumber between 1600 and 1500 cm^−1^ represents the amide I and amide II of proteins, the peaks were only present in the cheese samples which justified that the DSBCs do not have the amid group; this region in our study was observe at interval of 1624–1523 cm^−1^ corresponding to the cheese proteins. The C–H bends, C–O and C–N bonds have higher prominent peaks in DSBC extract at wavenumbers ranging from 1482 to 1000 (Doldolova et al. 2021). Other noticeable stretches were observed in the fortified cheese between 1368 and 1157 cm^−1^ wavelength which represent the C–O bond (Carballo‐Meilan et al. 2014; Grasel et al. 2016). Overall, the FTIR spectra of the DSBCs and the fortified cheese samples revealed distinct profound peaks and bands representing individual structural uniqueness.

### 3.5 Antimicrobial Activity of CC

The ability of some microbes to survive harsh environmental conditions makes it difficult to control microbes in food. This has drawn the attention of the food industry to attain food safety in food products by incorporating natural additives from plant origin without compromising the nutritional and sensory properties of food. The incorporation of DSBC into cottage cheese formulations presents a promising natural strategy to reduce microbial contamination, including the risk of mycotoxin‐producing fungi. Date seeds are rich in phenolic compounds such as gallic acid, caffeic acid, and flavonoids, which have demonstrated significant antimicrobial properties. For instance, methanolic extracts of date pits have shown antibacterial activity against pathogens like *Escherichia coli* and *Staphylococcus aureus* (Selim et al. 2021). The DSBCs incorporated in cottage cheese were investigated against yeast and mold, coliforms, and total bacterial count, as shown in Table 4. There was no growth of yeast or mold in any of the freshly prepared cheese samples. During the 7‐day storage period, only the control cheese had 2.65 ± 0.13 Log cfu/g while in the 14‐day storage, the presence of yeast and mold was recorded in all the CC samples, with the exception of 20% CC. Similarly, the CC showed no coliform growth in the freshly prepared cheese; however, at Days 7 and 14, the coliform growth was observed in the control, 2.5%, and 5% FCC were 2.82 ± 0.11, 2.91 ± 0.10, 2.54 ± 0.01 and 3.28 ± 0.24, 3.03 ± 0.00, 3.89 ± 0.03 (log cfu/g), respectively for Day 7 and 14. The higher percentage of DSBC in the CC (10% and 20% formulations) showed no coliform growth on Days 7 and 14. In the total bacterial count of CC, the results revealed bacterial growth only in the control and 2.5% CC. Formulations with higher DSBCs inhibited microbial growth during storage, which could be due to the presence of antioxidant properties of DSBCs which have the ability to inhibit oxidative processes that often accompany microbial spoilage, thereby extending the shelf life of dairy products. The dual action of DSBC—antimicrobial and antioxidant can—be particularly effective in curbing the growth of spoilage organisms and mycotoxin producers in cottage cheese during storage. Incorporating DSBC not only leverages the functional properties of date seeds but also aligns with sustainable practices by valorizing an agro‐industrial byproduct. This approach offers a natural and health‐promoting alternative to synthetic preservatives in dairy products (Alkhalidy et al. 2023). The findings of this study align with growing concerns about microbial and mycotoxin contamination in dairy products, particularly in regions where exposure risks are elevated, as highlighted by recent assessments (Xiong et al. 2022). The incorporation of DSBC into cottage cheese presents a sustainable and effective strategy to enhance microbial safety during storage. Given the increasing demand for clean‐label food preservation methods, our work supports the use of DSBC not only as a functional fortifying agent but also as a natural antimicrobial, contributing to improved shelf life and consumer safety in dairy systems.

**TABLE 4:** Microbiological analysis of the enriched cottage cheese with DSBC during 14‐day cold storage period.

Storage period	Samples	Yeasts and molds	Coliforms	Total bacteria count
Fresh	Control	nd	nd	3.93 ± 0.01^a^
	2.5% DSBC	nd	nd	3.86 ± 0.02^a^
	5% DSBC	nd	nd	3.06 ± 0.02^b^
	10% DSBC	nd	nd	nd
	20% DSBC	nd	nd	nd
Day 7	Control	2.65 ± 0.13	2.82 ± 0.11^a^	3.84 ± 0.03^a^
	2.5% DSBC	nd	2.91 ± 0.10^a^	3.65 ± 0.11^a^
	5% DSBC	nd	2.54 ± 0.01^b^	nd
	10% DSBC	nd	nd	nd
	20% DSBC	nd	nd	nd
Day 14	Control	3.89 ± 0.74^a^	3.28 ± 0.24^b^	3.73 ± 0.48^a^
	2.5% DSBC	3.83 ± 0.01^a^	3.03 ± 0.00^b^	3.11 ± 0.15^b^
	5% DSBC	3.73 ± 0.05^a^	3.89 ± 0.03^a^	nd
	10% DSBC	2.51 ± 0.01^b^	nd	nd
	20% DSBC	nd	nd	nd

*Note:* The mean value represents (*n* = 3) ± SD. The lower‐case letters in each column represent the significant difference among the various samples (*p* value < 0.05).

Abbreviation: nd, not detected.

### 3.6 Protein Profile of FFC

**FIGURE 5:**
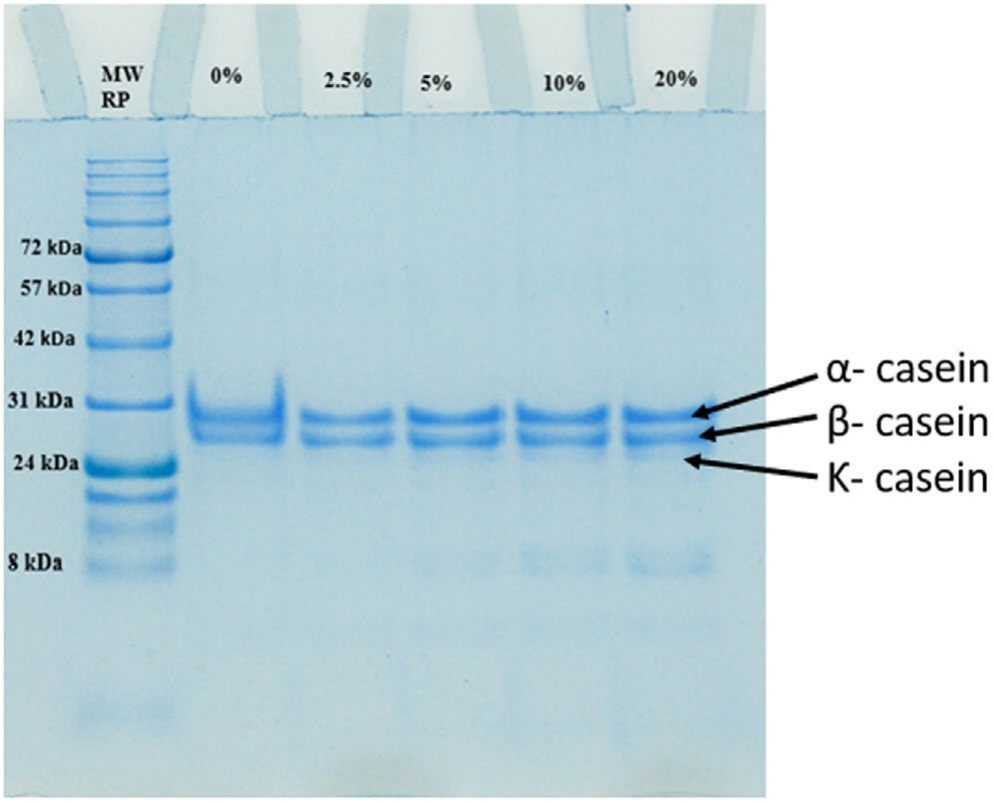
The SDS‐PAGE electropherograms of the various cottage cheese (CC) samples fortified with different levels (0% to 20%) of DSBC. MWRP represent the molecular weight of the reference protein.

SDS‐PAGE electropherograms of the control and DSBC‐fortified cheese formulations are shown in Figure 5. Cheese samples showed prominent bands for the α‐, β‐, and κ‐casein subunits. Cheese samples fortified with different levels of DSBC showed a slight shift and increase in the band intensity of casein proteins, an indication of complexation in polyphenolic compounds with casein proteins. In addition, α‐lactalbumin (a major whey protein) became slightly visible in the cheese samples with > 5% DSBC, whereas it was not visible in the control and cheese formulations with 0% and 2.5% DSBC. The slightly visible band of α‐lactalbumin observed at higher concentrations of DSBC could be attributed to the binding of date seed polyphenols (at higher concentrations) to α‐lactalbumin. Overall, the protein profile of CC was not affected by DSBC incorporation.

## 4 Conclusions

The extraction of bioactive compounds from date seeds using green and sustainable processing technologies can help to achieve net zero in the protection of the environment, climate change, and sustainability, and also help in achieving a circular bioeconomy. The utilization of DSBCs as a functional food ingredient/additive has proven to be a feasible approach for formulating cottage cheese containing substantial TPC and demonstrating antioxidant and antimicrobial activities. The enrichment of CC with DSBCs not only boosts the cheese bioactive profile but also contributes to an extended shelf life, offering both health benefits and improved product longevity. This study revealed that DSBCs may not necessarily increase the macronutrient content (like carbohydrates, proteins, or fats) of the cheese; however, the bioactive compounds add valuable micronutrients that enhance the CC overall nutritional profile, particularly its ability to offer health benefits beyond basic nutrition and also serve as a natural additive which extended the shelf life of the enriched CC.

## Author Contributions

**Jennifer Osamede Airouyuwa:** conceptualization (equal), data curation (lead), formal analysis (lead), methodology (lead), writing – original draft (lead). **Priti Mudgil:** methodology (equal), supervision (equal), validation (equal), writing – review and editing (equal). **Sajid Maqsood:** conceptualization (equal), project administration (lead), resources (lead), supervision (equal), validation (equal), writing – review and editing (lead).

## Conflicts of Interest

The authors declare no conflicts of interest.

## Supporting information


Data S1

## Data Availability

All data generated or analyzed during this study are included in this published article.
