# Supplementary material for: An Investigation Into Impact of Date Seed Bioactive Compound Addition on the Quality Attributes, Shelf Life, In Vitro Digestibility, and Bioactive Properties of Cottage Cheese
Source: Food Sci Nutr. 2025 Jul 4;13(7):e70535. doi: 10.1002/fsn3.70535 (PMC12227662; doi:10.1002/fsn3.70535)
Supplement: Supplementary file 1 — Data S1 [file FSN3-13-e70535-s001.docx]

# Supplementary Material

# *S.1.1 Materials and Methods Microwave Assisted Extraction (MAE)*

# The date seed bioactive compounds (DSBCs) were extracted based on the previously optimization condition of MAE (Airouyuwa, et al., 2023a). Briefly, 1 g of DSP were dissolved in 30 mL of 63% NADES and extracted using microwave assisted extraction at a temperature of 80 °C, 0.6 KW power for 5 min. Upon completion of the extraction process, the sample was centrifuge for 15 min at 10,000 g, and at a temperature of 4 °C; the supernatant was further stored at -20 °C. In addition, the extract undergoes further purification process by adjusting the pH to near neutral using 5M sodium hydroxide and then the extract was dialyzed for 12 hr to get rid of the NADES using a dialysis bag of 3.5 KDa. The dialyzed DSBC was concentrated using freeze dryer (Zirbus, Germany) and used in the formulation of CC. One of the ways to ascertain the removal of the NADES, is in the analysis of TPC. The presence of choline chloride and lactic acid in Folin-Ciocalteu reagent gives a creaming yellow coloration when determining the TPC of date seed extract. However, with the absent of choline chloride and lactic acid in the dialysed extract resulted in a bright yellow coloration which is the normal coloration when conventional solvents were used in the extraction of date seeds.

*S 1.2. Determination of total phenolic content (TPC)*

The TPC of the DSBC and CC samples were determined following the method described by Olatunde et al. (2018) with slight modifications. Briefly, 20 μL of a known concentration of the date seed extract was pipetted in a 96-well microplate. To each well, 10% Folin-Ciocalteu reagent of 150 μL was pipetted and vortex. After 5 min, 150 μL of 6% Na_2_CO_3_ was added to the solution. The solutions were incubated for 1 h at 37 ℃ and the absorbance was taken at 760 nm in a spectrophotometer microplate reader (Multiskan Sky, Thermo Fisher Scientific, Cambridge, MA, USA). The reference standard concentrations range from was (0-200 µg/ml) gallic acid. The TPC results were expressed in mg gallic acid equivalent per gram date seed powder (mg GAE/g DSP) and mg gallic acid equivalent per 100 grams date seed powder (mg GAE/100 g DSP) for DSBCs and the CC respectively.

*S1.3 Determination of antioxidant activity*

The radical scavenging activity 1,1-diphenyl-2-picrylhydrazyl (DPPH) of the DSBCs and CC samples were conducted as described by Mostafa et al., (2022). Briefly, 15 mM DPPH was prepared in methanolic solution, and 100 μL of the DPPH solution was added to 100 μL of the extract. The decrease in the absorbance was measured at 517 nm after 30 min with a spectrophotometer microplate reader (Multiskan Sky, Thermo Fisher Scientific, Cambridge, MA, USA). The results were expressed in mmol Trolox equivalents per gram of DSP (mmol TE/ g powder). The findings were reported in milli molars (mM) trolox equivalents per gram of DSP (mM TE/g DSP) for DSBCs and milli molars (mM) trolox equivalents per 100 grams of DSP (mM TE/g DSP) for CC.

The ferric reducing antioxidant power (FRAP) assay was analyzed by the method of Olatunde et al., (2018). The freshly prepared working FRAP solution containing 2.5 mL of ferric chloride (20 mmol), 25 mL of sodium acetate buffer (300 mmol, pH = 3.6), and 2.5 mL of TPTZ (10 mmol in 40 mmol hydrochloric acid), incubated at 37 ℃ for 30 min. The 285 μL FRAP working solution was added to 15 μL of the extract. After 30 min, the absorbance was measured at 593 nm. The Trolox equivalents (mmol) were obtained using standard concentration of Trolox (0-800 µm/ml).

*S1.4 Identification and quantification of the major phenolic compounds in date seed powder and Cottage cheese samples*

The analyses were performed using a Dionex UHPLC Ultimate-3600 (Thermo Scientific, USA), which includes a binary pump, an auto-sampler, a diode array detector, and a PDA detector. We conducted the separation on a Kinetex Phenyl-Hexyl column (150 mm × 2.1 mm i.e., 2.6 μm particle size) with a Phenyl guard column (2.1 × 4.6 mm). The column was thermostated at 50 °C, and the injection volume for all samples was 20 μL. The solvent gradient consisted of two phases: (A) water/phosphoric acid (99.5/0.5, v/v) and (B) acetonitrile/water/phosphoric acid (50/49.5/0.5, v/v/v). The gradient program was as follows: from 0 to 1 min, B (3%); from 1 to 16 min, B (50%); from 16 to 16.1 min, B (3%); and from 16.1 to 18.0 min, B (3%). For the detection and quantification of the compounds, chromatograms were recorded at 275 nm using the diode array detector. The software used for analysis was Chromeleon® Dionex version 7.2.4.817.

*S1.5* *In vitro simulated gastro-intestinal digestion (SGID) of cottage cheese*

Prior to the digestion of CC, 200 mL the various electrolytes comprising of simulated salivary fluid (SSF), simulated gastric fluid (SGF) and simulated intestinal fluid (SIF) stock solution at pH of 7, 3 and 7, respectively; were prepared by method of (Brodkorb et al., 2019). The simulated salivary fluid (SSF), simulated gastric fluid (SGF) and simulated intestinal fluid (SIF) stock solution at pH of 7, 3 and 7, respectively. These electrolytes were prepared measured as shown in table below and make-up to 200 mL with distilled water. In the Oral phase, 5 g of smashed CC was added to 5 ml of SSF including 75 U/mL of α-amylase and 1.5 M calcium chloride dihydrate (CaCl_2_(H_2_O)_2_) solution. The oral digestion was carried out while shaking the flask at 200 rpm while maintaining a temperature of 37 °C for 2 minutes. At the gastric phase, the oral bolus was transferred into 10 mL of SGF electrolyte including 2000 U/mL porcine pepsin, 60 U/mL gastric lipase, and 0.15 mM of (CaCl_2_(H_2_O)_2_). The gastric digestion was incubated for 2 h at 37 °C. After the gastric phase, 10 mL of the sample was withdrawn for analyses relating to gastric digestion and the remaining gastric chyme was transferred into 10 mL of SIF electrolyte including 100 U/mL of trypsin, 10 mM Bile salt and 0.6Mm (CaCl_2_(H_2_O)_2_). The intestinal digestion was incubated for 2 h at 37 °C at 200 rpm. After the digestion, the samples obtained before digestion, at the gastric phase and intestinal phase were used in the analysi of TPC, DPPH, FRAP and UHPLC profile.

The preparation of electrolytes for In-vitro simulated gastro-intestinal digestion (SGID) of CC

| **Salt solution** | **SSF (pH 7) in 200 mL** | **SGF (pH 3) in 200 mL** | **SIF (pH 7) in 200 mL** |
| --- | --- | --- | --- |
| KCl | 7.55 | 3.45 | 3.4 |
| KH_2_PO_4_ | 1.85 | 0.45 | 0.4 |
| NaHCO_3_ | 3.4 | 6.25 | 42.5 |
| NaCl | 0 | 5.9 | 19.2 |
| MgCl (H_2_O)_6_ | 0.25 | 0.2 | 0.165 |
| (NH_4_)_2_CO_3_ | 0.03 | 0.25 | 0 |
| HCl | 0.045 | 0.65 | 4.2 |
| CaCl_2_ (H_2_O)_2_ | 0.01 | 0.0025 | 0.3 |

CaCl_2_(H_2_O)_2_ should be added immediately before use.

*SI.6 Textural properties of CC*

The texture analysis of CC was carried out following the methods reported by (Jia et al., 2022) with slight modifications. The texture profile of CC was analyzed based on compression testing protocol using CT3 Texture Analyzer (Brookfield Middleboro, USA) equipped with a cylindrical probe (TA-AACC36) and a load cell of 4500g. Each CC was subjected to a single cycle compression with the following treatment; trigger force of 6.8 g, test speed 1mm/s, return speed of 1 mm/s, with a target of 4 mm. The parameters including hardness, adhesiveness, stringiness and resilience of the samples were recorded from the equipment software (Texture Expert for Windows, version 1.19).

*S1.7 Rheological properties of cottage cheese*

The rheological properties of the CC enriched with DSBC were evaluated using a Discovery Hybrid Rheometer (TA Instruments, Delaware, USA) according to the method of Azarashkan et al., 2022b), with slight modification. The parameters investigated includes complex viscosity (Pa.s), storage modulus (G’), and loss modulus (G’’). The cheese samples were sliced to a diameter of approximately 1 mm from the center of the cheese, placed in a polyethylene bag and equilibrate at room temperature for 3 hours before carrying out the analysis. The sliced piece of cheese sample was then placed on the parallel plate geometry with a diameter of 40 mm, a loading gap of 1000 µm, and a soak time of 600 s. The linear viscoelastic range was evaluated by a frequency sweep test at 0.1 – 100 Hz at 20 points intervals, and a percentage strain value of 1.0%.

*S1.8 Sodium dodecyl sulphate-polyacrylamide gel electrophoresis (SDS-PAGE) of cottage cheese*

SDS PAGE of CC was carried out by following a previous method described (Mudgil et al., 2019) with slight modification under the reducing conditions of 12% resolving gel and a 4% stacking gel. An aliquot of sample solution was mixed with sample buffer in a ratio of 1:6 v/v and boiled for 5 min. A 5 µL of the obtained sample was loaded in each well. A mini-Protean III apparatus (Bio-Rad, gel size 7 × 8 cm x 0.75 mm) was utilized for the electrophoresis experiment. Staining of the gel was implemented with 0.05% (w/v) of Coomassie Brilliant Blue R-250 for 4 h, followed by two steps of destaining processes using the destaining I solution (200 mL methanol, 30 mL acetic acid and 170 mL distilled water) and destaining II solution (50 mL methanol, 75 mL acetic acid and 75 mL distilled water).

**Table S2.1**: Total phenolic compounds and antioxidant activity (DPPH radical scavenging activity and FRAP antioxidant activity)

| Date seed | **TPC (mg GAE/g DSP)** | **DPPH (mM TE/g DSP)** | **FRAP (mM TE/g DSP)** |
| --- | --- | --- | --- |
|  | 153.58±72 | 601.89±83 | 787.53±45 |
